# Hospital Construction

**Published:** 1896-09-26

**Authors:** 


### HOSPITAL CONSTRUCTION.

### THE TORONTO GENERAL HOSPITAL.

#### The Buildings.

The General Hospital, Toronto, is an imposing-looking building, or, rather, group of buildings, for, with additions and extensions, there are no less than six separate blocks, containing in all four hundred beds. The hospital is built of white brick, in Old English style, four storeys in height, with five towers, the central one, from which there is a splendid view of the city, Lake Ontario, and many miles of surrounding country, being nearly a hundred feet high. The central block, with three wings running north, is the original hospital as built in 1854, though enlarged and improved since then. The western division is next in date, having been erected in 1877, and this part is now used as the nurses' home. The eastern block contains the Andrew Mercer Eye and Ear Infirmary and the medical superintendent's residence; both these wings are connected with the main building by corridors.

A continuous basement runs under these divided buildings connected by semi-underground tunnels with the central block, and here are kitchens and offices, servants' dining-rooms and bed-rooms, and all the usual storage rooms and offices. The general entrance is under the central tower, and leads into a hall from which ascends the central stairway. Close by is the passenger lift, the largest in Toronto, and capable of conveying patients on bed or stretcher. The corridors connecting the various blocks are wide, and open on verandahs. On the ground floor are the offices of the medical superintendent, Dr. Charles O'Reilly, and of the lady superintendent, Miss Snively; in the two wings running north are, in the one emergency wards and private wards; in the other, linen rooms, the medical assistants' dining-room, and private wards. The westernmost block is devoted to the nurses' quarters, while that on the east is, as already stated, the eye and ear infirmary. The second and third floors are divided into male and female medical and surgical wards, and the fourth floor is devoted to cases of contagious disease, and is reached by separate staircases. The central wing of the main building contains the clinical and operation theatres, out-patients' department, and laboratories, and to this portion of the hospital an addition is now being made, giving more room for out-patients, examination rooms, laboratories, and so forth. Behind the central division of the hospital are the laundry, disinfecting rooms, and mortuary. Separate buildings are the Burnside Lying-in Hospital, and the pavilion for women.

The hospital is lighted with gas and heated by steam and hot water. Most elaborate protection is made against fire, what with fire escapes, hydrants, an unlimited water supply, and, best of all, ample exit provision. Oil lamps are not used in the wards, old-fashioned candlesticks being considered safer for the use of night nurses.

#### History of the Hospital.

It was at the close of the war of 1812-14 that steps were taken to build a hospital in Toronto, a sum of £4,000 being given towards that end by the Loyal and Patriotic Society, the amount being the balance of funds collected for the succour of the widows and orphans of those killed in the war. In 1819 a hospital was built on a site bounded by King, John, Peter, and Adelaide Streets, but it was not used for its intended purpose till 1829, when it was opened for the reception of patients. Later on, the site having become very valuable, it was decided to erect a more commodious building in a quieter part of the town, and in 1854-5 the central block of the present hospital was built after plans by Mr. William Hay. In 1868, owing to lack of funds, the hospital was closed, and remained so for a year, at the end of which time the management was entirely reconstituted, and in the succeeding years kind friends came to its aid and improvements and alterations were gradually effected. In 1878, upon the advice of the Government Inspector that the various city charities should be amalgamated under one management, the Burnside Lying-in Hospital and the

Andrew Mercer Eye and Ear Infirmary were erected in the hospital grounds as portions of the General Hospital. In 1882 "The Pavilion" for diseases of women was added.

MEDICAL EDUCATION.

The Toronto General Hospital has always been to the fore in the encouragement of medical instruction and research, and many generations of students have owed much to the experience gained within its wards. There are three medical schools in Toronto, the University of Toronto Medical College, the Trinity Medical College, and the Women's Medical College, all having a large attendance of students, most of whom are attached to the General Hospital. Regular clinical lectures are given daily in the wards and in the large theatre, which is capable of seating six hundred students. Saturday afternoon is the time specially appointed for operations. Eight resident medical assistants are appointed annually, being elected from among the graduates each spring.

NURSES' TRAINING SCHOOL.

The Training School for Nurses attached to the Toronto General Hospital, of which Miss M. A. Sniveley is the able superintendent, was first opened in 1881. The staff at present, according to Miss Sniveley's recent report, numbers fifty-six pupil nurses, four graduate nurses, and a class of twenty-three graduates, these latter having received their certificates and badges in January last. The term of training is two years. Lectures are given by the medical and surgical staff during nine months of each year, and examinations are held every six months. Scattered over the world are now some 230 nurses who hold the certificate of the school, some in America, some in Europe, in Asia, and in Africa. Examinations are thus conducted: Nurses are required to pass an entrance examination in ordinary English and practical work; a written examination at the end of the first year, set by the superintendent of the school; and at the end of the second year a written and an oral examination before an examining board. Miss Sniveley has done much since her appointment as superintendent to carry out improvements and increase the practical efficiency of the training offered to nurses at this school. Especially are nurses taught to realise the "wide difference between the knowledge necessary for a doctor and that necessary for a nurse," and that her mission is to give intelligent and skilful co-operation to the medical profession in the care of the sick. The Toronto Training School is under the control of the trustees of the hospital, the medical superintendent having the general supervision, and the superintendent of the training school immediate control over the whole nursing department.

